# Prevalence and genotype distribution of human papillomavirus among 15,224 gynecological outpatients in Xuzhou, China, during 2023–2025

**DOI:** 10.3389/fpubh.2026.1969653

**Published:** 2026-10-05

**Authors:** Shuo Chai, Lulu Hu, Wenting Fan, Ying Li, Zhijian Liu, Ziwei Song, Shibao Li, Yingxing Zhu

**Affiliations:** 1 Department of Laboratory Medicine, Affiliated Hospital of Xuzhou Medical University, Xuzhou, China; 2 Department of Pathology, Affiliated Hospital of Xuzhou Medical University, Xuzhou, China

**Keywords:** cervical cancer, China, genotype distribution, human papillomavirus, prevalence, Xuzhou

## Abstract

**Objective:**

This study investigated HPV prevalence and genotype distribution among gynecological outpatients at the Affiliated Hospital of Xuzhou Medical University, aiming to inform region-specific cervical cancer prevention strategies.

**Methods:**

We determined the HPV subtypes by polymerase chain reaction (PCR) and analyzed the HPV prevalence, genotype distribution in 15,224 gynecological outpatients by age group and year.

**Results:**

Among the 15,224 outpatients, 4,884 (32.08%) tested positive for HPV, with single high-risk HPV (HR-HPV) infection being the predominant pattern (16.48%). The top five HR-HPV genotypes were HPV52 (5.58%), 16 (4.93%), 58 (4.49%), 53 (3.54%), and 56 (2.51%). The top three low-risk HPV (LR-HPV) genotypes were HPV42 (3.30%), 81 (2.16%), and 43 (1.76%). From 2023 to 2025, the overall HPV prevalence among this study population exhibited a significant increasing trend, rising from 27.57 to 36.63% (*p(linear by linear) < 0.001*). Overall HPV prevalence differed significantly across age groups (*p < 0.001*), exhibiting a bimodal distribution, with the highest rate in the ≤24 years group (42.30%), followed by the 55–64 years group (37.29%) and the ≥65 years group (36.43%), and the lowest in the 25–34 years group (29.05%). Across age groups, the top five HR-HPV genotypes varied slightly but consistently included HPV52, 16, 58, 53, and 56. For LR-HPV infections, the top three genotypes were HPV6, 42, and 11 in the ≤24 years group, whereas HPV42, 81, and 43 ranked as the top three in all other age groups.

**Conclusion:**

The prevalence of HPV infection among women attending gynecological outpatient clinics of the Affiliated Hospital of Xuzhou Medical University is relatively high and has demonstrated an increasing trend over the study period. Adolescents and middle-aged and older women exhibit higher HPV prevalence. The predominant genotypes are HR-HPV52, 16, 58, 53, and 56, as well as LR-HPV42, 81, and 43. In addition to strengthening universal health education, the observed age-specific distribution suggests that young women and middle-aged and older women may be important target groups for future prevention strategies. Furthermore, the high prevalence of non-vaccine-covered genotypes (HPV53, 56, 42, 81, and 43) warrants continued surveillance and further investigation of their clinical significance, although these implications should be considered preliminary.

## Introduction

Human papillomavirus (HPV) is an epitheliotropic double-stranded DNA virus, with over 200 genotypes having been identified to date. Based on their oncogenic potential, these genotypes are categorized as HR-HPV and LR-HPV types. The high-risk types predominantly include HPV16, 18, 31, 33, 35, 39, 45, 51, 52, 56, 58, 68, among which HPV16 and HPV18 are the most prevalent high-risk genotypes worldwide (1). HPV is primarily transmitted through sexual contact, and although the majority of women are at risk for infection during their lifetime, most infections are transient and can be effectively cleared by the host immune system (2).

Extensive epidemiological and molecular biological studies have established that persistent infection with HR-HPV is a necessary condition for the development of cervical cancer and its precursor lesions. Upon integration of the viral genome into the host cell DNA, disruption of the viral E2 gene occurs, leading to the release of E6 and E7 oncogenes from repression and their subsequent overexpression. The E6 protein degrades the tumor suppressor p53, while the E7 protein binds and inactivates the retinoblastoma protein (Rb); together, these events disrupt host cell cycle regulation and apoptotic pathways, ultimately driving malignant transformation (3). Cervical cancer currently ranks as the fourth most common malignancy among women worldwide. According to GLOBOCAN 2022 estimates, there are approximately 660,000 new cases and 340,000 deaths annually, with the disease burden being particularly heavy in low- and middle-income countries, which account for about 90% of global deaths; this burden persists even though cervical cancer has a well-defined etiology and is theoretically preventable (4).

To accelerate the global elimination of cervical cancer, the World Health Organization (WHO) launched the “Global Strategy to Accelerate the Elimination of Cervical Cancer” in 2020, proposing the “90–70–90” targets: by 2030, 90% of girls should be fully vaccinated with HPV vaccine by age 15, 70% of women should receive high-performance screening at ages 35 and 45, and 90% of women identified with cervical precancerous lesions and cervical cancer should have access to appropriate treatment. However, global HPV vaccination coverage remains suboptimal. An analysis of data from 194 WHO member states has reported that in 2023, the weighted average coverage of the first dose and full-course of HPV vaccination among girls aged 9–14 years was only 61.6 and 47.6%, respectively (5), far below the WHO target of 90%. In China, vaccination uptake has gradually increased since HPV vaccines were licensed in 2016, but significant disparities persist between urban and rural areas and across different regions in terms of vaccine accessibility and screening coverage, influenced by socioeconomic status, educational level, and healthcare availability (6). Therefore, given that current prevention and treatment strategies have not yet achieved universal coverage, continuous regional epidemiological surveillance of HPV infection remains critical for optimizing vaccination strategies and resource allocation for screening efforts.

Both HPV prevalence and the distribution of dominant genotypes exhibit marked geographical and population-specific variations. A large-scale pooled analysis involving 2,728,321 Chinese women has demonstrated that the overall HPV prevalence among Chinese women ranges from 13.1 to 18.8%, with the highest rates observed in South China and Northeast China, and the lowest in Northwest China. Regarding genotype distribution, HPV52, HPV16, and HPV58 are the most common types among Chinese women, a pattern that diverges from the global predominance of HPV16/18 (7). Moreover, even within the same province, different cities may show distinct dominant genotypes (8). HPV prevalence also exhibits a bimodal age-specific distribution, with two peaks typically observed in women aged ≤25 years and ≥46 years (9). These findings underscore the need for region- and population-specific current data to inform precision prevention strategies.

Xuzhou, located in the northern part of Jiangsu Province, China, serves as a central city in the Huaihai Economic Zone, with a large and mobile population. To date, however, large-scale, systematic studies on HPV infection among women attending gynecological outpatient clinics in this region remain limited. To address this gap, the present study retrospectively analyzed HPV testing data from 15,224 gynecological outpatients in the gynecological department during the period 2023–2025. The study aimed to systematically describe the overall HPV prevalence, genotype distribution, and their variations across different years and age subgroups in this population, thereby contributing to the regional epidemiological knowledge base for HPV infection.

## Materials and methods

### Study population

This retrospective study enrolled women who underwent HPV testing at the gynecological outpatient clinic of the Affiliated Hospital of Xuzhou Medical University between January 1, 2023, and December 31, 2025. All participants were required to meet the following inclusion criteria: (1) female patients attending the gynecological outpatient clinic; (2) residents of Xuzhou; (3) sexually active. The exclusion criteria were: (1) incomplete clinical information or test results; (2) for patients with multiple visits, only the first test result was retained; (3) use of vaginal medications or douching within 3 days before specimen collection; (4) sexual intercourse within 24 h before specimen collection; (5) menstruation or pregnancy; (6) history of hysterectomy. All participants satisfied all inclusion criteria and none of the exclusion criteria. Ultimately, 15,224 patients were enrolled in the study, with ages ranging from 15 to 94 years (mean age: 44.7 ± 11.9 years). A flow diagram of participant selection is presented in Supplementary Figure S1. This study was conducted in accordance with the Declaration of Helsinki and was approved by the Ethics Committee of the Affiliated Hospital of Xuzhou Medical University (ethics approval number: XYFY2025-KL505-01).

### Sample collection and HPV DNA detection

Gynecologists collected cervical exfoliated cell specimens from each patient in the lithotomy position. After exposing the cervix with a sterile speculum and clearing surface secretions with a sterile cotton swab, a dedicated HPV sampling brush was inserted into the cervical os and rotated clockwise five to six times at the squamocolumnar junction. The brush head was placed into a tube with preservation solution, the handle was broken off, and the tube was capped, labeled, stored at 2–8 °C, and transported to the laboratory within 24 h. HPV DNA genotyping was performed using the Human Papillomavirus (HPV) Nucleic Acid Detection Kit (Fluorescence PCR), manufactured by Xiamen Amplly Biotechnology Co., Ltd. (NMPA registration number: 20163400699), which identifies 24 HPV genotypes. The classification of HPV genotypes into high-risk and low-risk groups followed the manufacturer’s instructions. This grouping is informed by the IARC Monographs on the carcinogenicity of HPV: the high-risk group comprises genotypes classified by IARC as Group 1, 2A, or 2B, and the low-risk group includes genotypes classified as Group 3 or not formally evaluated by IARC. Accordingly, 17 genotypes were classified as high-risk (HPV16, 18, 31, 33, 35, 39, 45, 51, 52, 53, 56, 58, 59, 66, 68, 73, and 82) and 7 as low-risk (HPV6, 11, 42, 43, 44, 81, and 83). The assay had been validated in our laboratory according to the manufacturer’s protocol and CLSI guidelines prior to clinical use. The manufacturer reports an analytical sensitivity of 500 copies/mL for all 24 genotypes and a specificity of 99.5% with no cross-reactivity. Inter- and intra-assay coefficients of variation (CVs) for Ct values were <3.5% based on our laboratory’s internal quality control data. Specimens with Ct values between 38 and 39 were considered borderline and retested in duplicate; if the repeat remained borderline, the sample was considered indeterminate and excluded. Invalid results (internal control failure) were retested; if the repeat remained invalid, the sample was excluded. The PCR cycling conditions were as follows: an initial step at 38 °C for 5 min, denaturation at 95 °C for 2 min, followed by 40 cycles of denaturation at 95 °C for 15 s and annealing at 58 °C for 50 s, with a final hold at 4 °C. The assessment of HPV status was based on the observation of cycle threshold (Ct) values at which the fluorescent signal reaches the specified type-specific threshold. A Ct value of ≤39 indicated a positive HPV result, while a Ct value >39 was interpreted as negative. All reactions were performed on a SLAN-96S Real-Time PCR System (Shanghai Hongshi Medical Technology Co., Ltd., Shanghai, China) strictly according to the manufacturer’s instructions for the instrument. Positive and negative controls were used for each test, and the kit instructions were strictly followed.

### Statistical analysis

Data processing and statistical analyses were performed using Microsoft Excel 2021 and SPSS 26.0. Categorical variables were presented as *n* (%), genotype-specific prevalence was calculated as the number of women positive for a given HPV genotype divided by the total number of women tested (*N* = 15,224), expressed as a percentage, and comparisons between groups were made using the chi-square test. The linear-by-linear association test and gamma value were used to evaluate changes in HPV prevalence across the year groups. To assess whether the temporal trend was independent of age, multivariable logistic regression was performed with HPV positivity as the dependent variable and study year (reference: 2023) and age group (reference: 25–34 years) as categorical independent variables. Adjusted odds ratios (ORs) with 95% confidence intervals (CIs) were calculated. A two-sided *p* value <0.05 was considered statistically significant for general comparisons. For the 24 genotype-specific comparisons, the Bonferroni-corrected significance threshold was set at *p* < 0.0021 (0.05/24).

## Results

### Overall HPV prevalence and genotype distribution

Among the 15,224 women tested, 4,884 were HPV-positive, yielding an overall prevalence of 32.08%. Based on infection patterns according to the number of genotypes detected, single infections were identified in 3,187 women (20.93%), double infections in 1,048 (6.88%), triple infections in 387 (2.54%), quadruple infections in 160 (1.05%), and ≥quintuple infections in 102 (0.67%). Based on infection types defined by both genotype count and high−/low-risk combinations, the infections were categorized as follows: single high-risk infections in 2,509 women (16.48%), single low-risk infections in 678 (4.45%), multiple high-risk infections in 822 (5.40%), mixed high- and low- risk infections in 825 (5.42%), and multiple low-risk infections in 50 (0.33%). Among the positive samples, the top five genotypes by prevalence were HPV52 (5.58%), 16 (4.93%), 58 (4.49%), 53 (3.54%), and 42 (3.30%). The top five high-risk genotypes were HPV52 (5.58%), 16 (4.93%), 58 (4.49%), 53 (3.54%), and 56 (2.51%); the top three low-risk genotypes were HPV42 (3.30%), 81 (2.16%), and 43 (1.76%). The prevalence and infection patterns for each genotype are shown in Figure 1.

**Figure 1:**
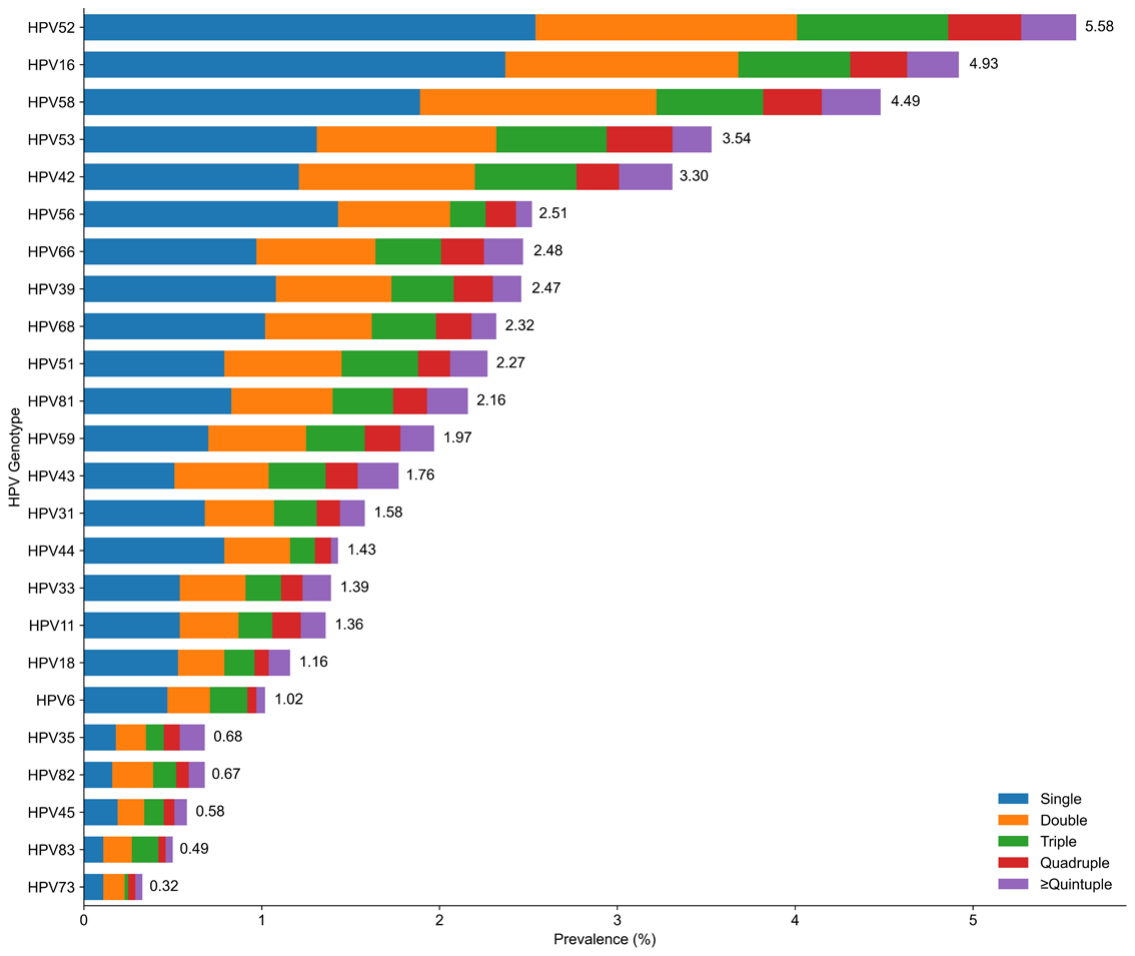
Prevalence and genotype distribution of HPV infections (including infection patterns).

### HPV prevalence and genotype distribution among different years

We compared the age distribution of participants across the 3 years from 2023 to 2025. The results showed that the age composition of outpatients remained relatively stable during the study period, with the 35–44 and 45–54 age groups consistently accounting for the largest proportions. The distribution of participants by age group and year is shown in Supplementary Table S1. A chi-square test of homogeneity revealed a statistically significant difference in the age-group composition of participants from 2023 to 2025 (*χ*^2^ = 73.506, *p* < 0.001). However, the effect size (Cramér’s *V* = 0.049) was very small and not of practical clinical significance. Chi-square tests showed that the overall HPV prevalence among women attending gynecological clinics differed significantly from 2023 to 2025 (*χ*^2^ *= 107.120, p < 0.001*). Stratified chi-square tests revealed that, except for multiple low-risk infections for which the differences were not statistically significant (*p > 0.05*), the differences in prevalence across all infection patterns (single to ≥quintuple genotypes) and infection types (single high-risk, single low-risk, multiple high-risk, and mixed high- and low-risk infections) were statistically significant (*all p < 0.05*). Linear-by-linear association tests indicated that the overall HPV prevalence among these women increased continuously from 2023 to 2025, rising from 27.57 to 36.63% (*χ*^2^*(linear by linear) = 101.825, p(linear by linear) < 0.001*). Similarly, stratified linear-by-linear association tests showed that, except for multiple low-risk infections, which exhibited a fluctuating pattern with an initial decrease followed by an increase and thus did not follow a linear trend, the prevalence rates of all infection patterns and infection types showed significant linear upward trends over the years (*all p(linear by linear) < 0.05*; Table 1). To assess whether the observed temporal increase was independent of age, a multivariable logistic regression analysis was performed with study year and age group as independent variables. After adjusting for age group, the odds of HPV positivity were significantly higher in 2024 (adjusted OR = 1.346, 95% CI: 1.240–1.462, *p* < 0.001) and 2025 (adjusted OR = 1.501, 95% CI: 1.380–1.632, *p* < 0.001) compared with 2023, indicating a continuous upward trend independent of age. The complete age-adjusted results for all infection patterns, types, and the eight significant genotypes are presented in Supplementary Table S2.

**Table 1:** Prevalence of HPV infection patterns and types in different year groups.

HPV infection	Positive cases	% (95% CI) for all samples	2023 (*n* = 6,286)	2024 (*n* = 4,671)	2025 (*n* = 4,267)	*χ* ^2^	*p*	*χ*^2^ (linear by linear)	*p* (linear by linear)	Gamma	Rate difference (95% CI)
Positive cases	4,884	32.08 (31.37–32.80)	1,733 (27.57)	1,588 (34.00)	1,563 (36.63)	107.120	<0.001	101.825	<0.001	0.145	9.06% (7.24–10.88)
Infection patterns											
Single	3,187	20.93 (20.51–21.36)	1,157 (18.41)	1,034 (22.14)	996 (23.34)	43.300	<0.001	40.215	<0.001	0.105	4.94% (3.35–6.53)
Double	1,048	6.88 (6.49–7.27)	363 (5.77)	354 (7.58)	331 (7.76)	20.659	<0.001	17.360	<0.001	0.111	1.98% (0.99–2.97)
Triple	387	2.54 (2.29–2.79)	134 (2.13)	124 (2.65)	129 (3.02)	8.499	0.014	8.421	0.004	0.122	0.89% (0.27–1.52)
Quadruple	160	1.05 (0.89–1.21)	48 (0.76)	43 (0.92)	69 (1.62)	18.904	<0.001	16.664	<0.001	0.255	0.85% (0.42–1.29)
≥Quintuple	102	0.67 (0.54–0.80)	31 (0.49)	33 (0.71)	38 (0.89)	6.166	0.046	6.156	0.013	0.199	0.40% (0.07–0.73)
Infection types											
Single HR	2,509	16.48 (15.89–17.07)	937 (14.91)	801 (17.15)	771 (18.07)	20.654	<0.001	19.638	<0.001	0.080	3.16% (1.71–4.62)
Single LR	678	4.45 (4.13–4.78)	220 (3.50)	233 (4.99)	225 (5.27)	23.309	<0.001	20.585	<0.001	0.147	1.77% (0.96–2.58)
HR + HR	822	5.40 (5.04–5.76)	293 (4.66)	260 (5.57)	269 (6.30)	13.801	0.001	13.757	<0.001	0.109	1.64% (0.75–2.54)
HR + LR	825	5.42 (5.06–5.78)	255 (4.06)	284 (6.08)	286 (6.70)	40.463	<0.001	37.399	<0.001	0.180	2.65% (1.75–3.54)
LR + LR	50	0.33 (0.24–0.42)	28 (0.45)	10 (0.21)	12 (0.28)	4.785	0.091	–	–	–	−0.16% (−0.39 to 0.07)

Rate difference = HPV prevalence in 2025 − HPV prevalence in 2023.

Over the 3 years, among the 17 HR-HPV genotypes, HPV52, 16, and 58 consistently ranked as the top three. After Bonferroni correction for multiple comparisons (corrected significance threshold: *p* < 0.0021), six HR-HPV genotypes (HPV52, 56, 39, 59, 18, and 68) showed statistically significant overall differences across the 3 years, all of which also exhibited significant monotonically increasing trends. Among the 7 LR-HPV genotypes during 2023–2025, HPV42 and 81 consistently ranked as the top two. Among LR-HPV genotypes, HPV43 and HPV11 showed significant overall differences, with HPV11 additionally showing a significant increasing trend. Although some other genotypes (e.g., HPV53, HPV58, HPV66, HPV31, HPV35, HPV42, and HPV44) showed *p* values < 0.05 in unadjusted analyses, these differences did not reach statistical significance after Bonferroni correction and should be interpreted with caution. The annual genotype-specific prevalence distributions are shown in Table 2 and Figure 2A.

**Table 2:** HPV genotype distribution in different year groups.

HPV genotype	Positive cases	% (95% CI) for all samples	2023 (*n* = 6,286)	2024 (*n* = 4,671)	2025 (*n* = 4,267)	*χ* ^2^	*p*	*χ*^2^ (linear by linear)	*p* (linear by linear)	Gamma	Rate difference (95% CI)
Positive cases	4,884	32.08 (31.37–32.80)	1,733 (27.57)	1,588 (34.00)	1,563 (36.63)	107.120	<0.001	101.825	<0.001	0.145	9.06% (7.24–10.88)
HR-HPV											
HPV52	850	5.58 (5.22–5.95)	301 (4.79)	263 (5.63)	286 (6.70)	17.695	<0.001	17.615	<0.001	0.120	1.92% (1.00–2.83)
HPV16	750	4.93 (4.58–5.27)	296 (4.71)	213 (4.56)	241 (5.65)	6.717	0.035	–	–	–	–
HPV58	683	4.49 (4.16–4.82)	251 (3.99)	217 (4.65)	215 (5.04)	6.885	0.032	6.759	0.009	0.084	–
HPV53	539	3.54 (3.25–3.83)	198 (3.15)	156 (3.34)	185 (4.34)	11.259	0.004	9.737	0.002	0.109	–
HPV56	382	2.51 (2.26–2.76)	118 (1.88)	135 (2.89)	129 (3.02)	17.644	<0.001	15.111	<0.001	0.166	1.15% (0.53–1.76)
HPV66	377	2.48 (2.23–2.72)	129 (2.05)	123 (2.63)	125 (2.93)	8.787	0.012	8.518	0.004	0.125	–
HPV39	376	2.47 (2.22–2.72)	119 (1.89)	125 (2.68)	132 (3.09)	16.395	<0.001	15.952	<0.001	0.170	1.20% (0.58–1.82)
HPV68	353	2.32 (2.08–2.56)	113 (1.80)	127 (2.72)	113 (2.65)	12.884	0.002	–	–	–	0.85% (0.27–1.43)
HPV51	346	2.27 (2.04–2.51)	122 (1.94)	110 (2.35)	114 (2.67)	6.317	0.043	6.283	0.012	0.111	–
HPV59	300	1.97 (1.75–2.19)	91 (1.45)	101 (2.16)	108 (2.53)	16.725	<0.001	16.23	<0.001	0.191	1.08% (0.53–1.64)
HPV31	240	1.58 (1.38–1.77)	75 (1.19)	93 (1.99)	72 (1.69)	11.465	0.003	–	–	–	–
HPV33	212	1.39 (1.21–1.58)	78 (1.24)	63 (1.35)	71 (1.66)	3.407	0.182	3.157	0.076	0.099	–
HPV18	177	1.16 (0.99–1.33)	58 (0.92)	45 (0.96)	74 (1.73)	16.896	<0.001	13.184	<0.001	0.215	0.81% (0.35–1.27)
HPV35	104	0.68 (0.55–0.81)	27 (0.43)	44 (0.94)	33 (0.77)	11.084	0.004	–	–	–	–
HPV82	102	0.67 (0.54–0.80)	53 (0.84)	30 (0.64)	19 (0.45)	6.124	0.047	6.123	0.013	−0.207	–
HPV45	89	0.58 (0.46–0.71)	29 (0.46)	25 (0.54)	35 (0.82)	5.916	0.052	5.302	0.021	0.194	–
HPV73	49	0.32 (0.23–0.41)	21 (0.33)	15 (0.32)	13 (0.30)	0.069	0.966	0.068	0.794	−0.031	–
LR-HPV											
HPV42	503	3.30 (3.02–3.59)	173 (2.75)	171 (3.66)	159 (3.73)	10.236	0.006	8.454	0.004	0.110	–
HPV81	329	2.16 (1.93–2.39)	127 (2.02)	105 (2.25)	97 (2.27)	1.009	0.604	0.855	0.355	0.043	–
HPV43	268	1.76 (1.55–1.97)	78 (1.24)	104 (2.23)	86 (2.02)	17.285	<0.001	–	–	–	0.77% (0.27–1.28)
HPV44	218	1.43 (1.24–1.62)	77 (1.22)	60 (1.28)	81 (1.90)	9.202	0.010	7.461	0.006	0.147	–
HPV11	207	1.36 (1.18–1.54)	38 (0.60)	73 (1.56)	96 (2.25)	53.373	<0.001	52.934	<0.001	0.403	1.65% (1.16–2.13)
HPV6	155	1.02 (0.86–1.18)	54 (0.86)	46 (0.98)	55 (1.29)	4.736	0.094	4.483	0.034	0.137	–
HPV83	75	0.49 (0.38–0.60)	29 (0.46)	30 (0.64)	16 (0.37)	3.464	0.177	–	–	–	–

For the 24 HPV genotypes compared across 3 years, the corrected significance threshold was set at *α* = 0.05/24 = 0.0021. Genotype-specific prevalence was calculated using the total number of women tested (*N* = 15,224) as the denominator. Rate difference = HPV prevalence in 2025 − HPV prevalence in 2023. For the remaining genotypes, comparisons did not reach the Bonferroni-corrected significance threshold (*p* < 0.0021); therefore, rate differences were not reported.

**Figure 2:**
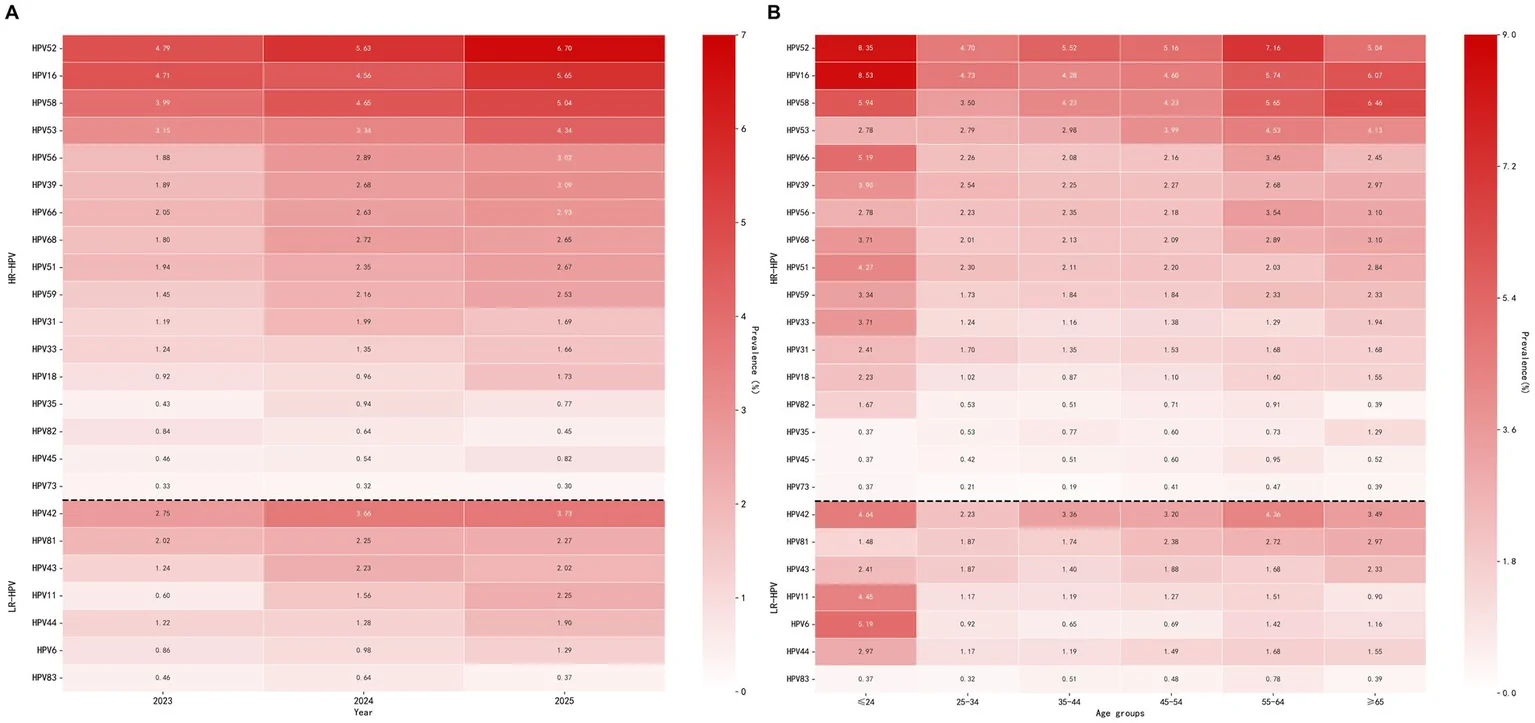
Annual and age-specific genotype distribution of HPV infections. **(A)** Genotype-specific prevalence by year (2023–2025). **(B)** Genotype-specific prevalence by age group.

### HPV prevalence and genotype distribution in different age groups

Patients were divided into six age groups: ≤24, 25–34, 35–44, 45–54, 55–64, and ≥65 years. The overall HPV prevalence differed significantly among age groups (*χ*^2^ *= 87.198, p < 0.001*), showing a bimodal distribution with an initial decrease followed by an increase. The highest prevalence was in the ≤24 years group (42.30%), followed by the 55–64 years group (37.29%) and the ≥65 years group (36.43%), and the lowest was in the 25–34 years group (29.05%). Multivariable logistic regression analysis showed that, after adjusting for study year, age remained an independent factor associated with HPV positivity. Compared with the 25–34 years group, the ≤24 years group (adjusted OR = 1.786, 95% CI: 1.477–2.160), the 55–64 years group (adjusted OR = 1.407, 95% CI: 1.251–1.582), and the ≥65 years group (adjusted OR = 1.368, 95% CI: 1.156–1.618) all had significantly higher odds of HPV positivity, confirming the bimodal age distribution. The prevalence of different infection patterns also differed significantly across age groups (*p < 0.05*), with multiple infections accounting for a higher proportion in the younger age groups. For infection types, comparisons across age groups showed significant differences for all types(all *p < 0.05*) except multiple low-risk infections (*p = 0.570*; Table 3; Figure 3).

**Table 3:** Prevalence of HPV infection patterns and types in different age groups.

HPV genotype	Positive cases	% (95% CI) for all samples	≤24 (*n* = 539)	25–34 (*n* = 2,830)	35–44 (*n* = 4,133)	45–54 (*n* = 4,631)	55–64 (*n* = 2,317)	≥65 (*n* = 774)	*χ2*	*p*	Rate difference (95% CI)
Positive cases	4,884	32.08 (31.37–32.80)	228 (42.30%)	822 (29.05%)	1,215 (29.40%)	1,473 (31.81%)	864 (37.29%)	282 (36.43%)	87.198	<0.001	13.26% (8.76–17.75)
Infection patterns											
Single	3,187	20.93 (20.29–21.58)	125 (23.19%)	551 (19.47%)	805 (19.48%)	1,001 (21.62%)	524 (22.62%)	181 (23.39%)	18.687	0.002	3.72% (−0.13 to 7.57)
Double	1,048	6.88 (6.49–7.27)	49 (9.09%)	176 (6.22%)	270 (6.53%)	302 (6.52%)	194 (8.37%)	57 (7.36%)	16.085	0.007	2.87% (0.29–5.46)
Triple	387	2.54 (2.29–2.79)	29 (5.38%)	61 (2.16%)	81 (1.96%)	95 (2.05%)	95 (4.10%)	26 (3.36%)	54.179	<0.001	3.23% (1.25–5.20)
Quadruple	160	1.05 (0.89–1.21)	12 (2.23%)	21 (0.74%)	38 (0.92%)	49 (1.06%)	30 (1.29%)	10 (1.29%)	12.204	0.032	1.48% (0.20–2.77)
≥Quintuple	102	0.67 (0.54–0.80)	13 (2.41%)	13 (0.46%)	21 (0.51%)	26 (0.56%)	21 (0.91%)	8 (1.03%)	32.391	<0.001	1.95% (0.63–3.27)
Infection types											
Single HR	2,509	16.48 (15.89–17.07)	87 (16.14%)	439 (15.51%)	639 (15.46%)	782 (16.89%)	413 (17.82%)	149 (19.25%)	13.004	0.023	0.63% (−2.75 to 4.01)
Single LR	678	4.45 (4.13–4.78)	38 (7.05%)	112 (3.96%)	166 (4.02%)	219 (4.73%)	111 (4.79%)	32 (4.13%)	13.662	0.018	3.09% (0.81–5.37)
HR + HR	822	5.40 (5.04–5.76)	45 (8.35%)	140 (4.95%)	214 (5.18%)	219 (4.73%)	159 (6.86%)	45 (5.81%)	24.754	<0.001	3.40% (0.93–5.87)
HR + LR	825	5.42 (5.06–5.78)	56 (10.39%)	126 (4.45%)	181 (4.38%)	239 (5.16%)	171 (7.38%)	52 (6.72%)	60.397	<0.001	5.94% (3.25–8.62)
LR + LR	50	0.33 (0.24–0.42)	2 (0.37%)	5 (0.18%)	15 (0.36%)	14 (0.30%)	10 (0.43%)	4 (0.52%)	3.860	0.570	0.19% (−0.34 to 0.73)

Rate difference = HPV prevalence in the ≤24 years group − HPV prevalence in the 25–34 years group.

**Figure 3:**
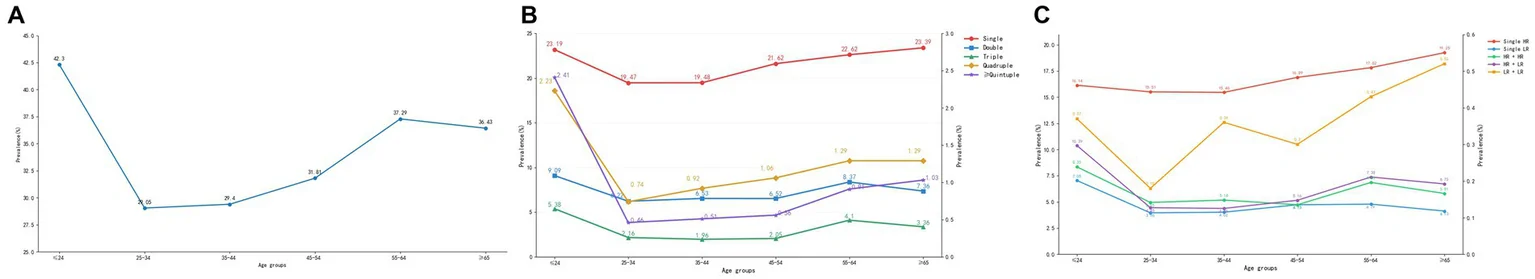
Prevalence of HPV infection patterns and types by age group. **(A)** Overall HPV prevalence; **(B)** infection patterns (single, double, triple, quadruple, and ≥quintuple); **(C)** infection types (single HR, single LR, HR+HR, HR+LR, and LR+LR).

Among the 17 HR-HPV genotypes, the top five predominant genotypes in each age group fluctuated slightly in rank but mostly remained HPV52, 16, 58, 53, and 56. After Bonferroni correction for multiple comparisons (corrected significance threshold: *p* < 0.0021), six HR-HPV genotypes (HPV52, 16, 58, 66, 33, and 53) showed statistically significant differences across age groups. Among the 7 LR-HPV genotypes, except for the ≤24 years group, in which the top three predominant genotypes were HPV6, 42, and 11, the top three in the other age groups were HPV42, 81, and 43. HPV6 and 11 had the highest prevalence in the ≤24 years group (5.19 and 4.45%, respectively). Among LR-HPV genotypes, HPV11, HPV6, and HPV42 showed significant age-related differences. Although some other genotypes (e.g., HPV68, HPV18, and HPV82) showed *p* values < 0.05 in unadjusted analyses, these differences did not reach statistical significance after Bonferroni correction and should be interpreted with caution (Table 4; Figure 2B).

**Table 4:** HPV genotype distribution in different age groups.

HPV genotype	Positive cases	% (95% CI) for all samples	≤24 (*n* = 539)	25–34 (*n* = 2,830)	35–44 (*n* = 4,133)	45–54 (*n* = 4,631)	55–64 (*n* = 2,317)	≥65 (*n* = 774)	*χ* ^2^	*p*	Rate difference (95% CI)
Positive cases	4,884	32.08 (31.37–32.80)	228 (42.30%)	822 (29.05%)	1,215 (29.40%)	1,473 (31.81%)	864 (37.29%)	282 (36.43%)	87.198	<0.001	13.26% (8.76–17.75)
HR-HPV											
HPV52	850	5.58 (5.22–5.95)	45 (8.35)	133 (4.70)	228 (5.52)	239 (5.16)	166 (7.16)	39 (5.04)	25.038	<0.001	3.65% (1.19–6.11)
HPV16	750	4.93 (4.58–5.27)	46 (8.53)	134 (4.73)	177 (4.28)	213 (4.60)	133 (5.74)	47 (6.07)	25.362	<0.001	3.80% (1.31–6.28)
HPV58	683	4.49 (4.16–4.82)	32 (5.94)	99 (3.50)	175 (4.23)	196 (4.23)	131 (5.65)	50 (6.46)	24.812	<0.001	2.44% (0.33–4.55)
HPV53	539	3.54 (3.25–3.83)	15 (2.78)	79 (2.79)	123 (2.98)	185 (3.99)	105 (4.53)	32 (4.13)	19.674	0.001	−0.01% (−1.53–1.51)
HPV56	382	2.51 (2.26–2.76)	15 (2.78)	63 (2.23)	97 (2.35)	101 (2.18)	82 (3.54)	24 (3.10)	14.730	0.012	–
HPV66	377	2.48 (2.23–2.72)	28 (5.19)	64 (2.26)	86 (2.08)	100 (2.16)	80 (3.45)	19 (2.45)	30.787	<0.001	2.93% (0.98–4.89)
HPV39	376	2.47 (2.22–2.72)	21 (3.90)	72 (2.54)	93 (2.25)	105 (2.27)	62 (2.68)	23 (2.97)	7.450	0.189	–
HPV68	353	2.32 (2.08–2.56)	20 (3.71)	57 (2.01)	88 (2.13)	97 (2.09)	67 (2.89)	24 (3.10)	12.900	0.024	–
HPV51	346	2.27 (2.04–2.51)	23 (4.27)	65 (2.30)	87 (2.11)	102 (2.20)	47 (2.03)	22 (2.84)	12.040	0.034	–
HPV59	300	1.97 (1.75–2.19)	18 (3.34)	49 (1.73)	76 (1.84)	85 (1.84)	54 (2.33)	18 (2.33)	8.935	0.112	–
HPV31	240	1.58 (1.38–1.77)	13 (2.41)	48 (1.70)	56 (1.35)	71 (1.53)	39 (1.68)	13 (1.68)	4.272	0.511	–
HPV33	212	1.39 (1.21–1.58)	20 (3.71)	35 (1.24)	48 (1.16)	64 (1.38)	30 (1.29)	15 (1.94)	25.042	<0.001	2.47% (0.83–4.12)
HPV18	177	1.16 (0.99–1.33)	12 (2.23)	29 (1.02)	36 (0.87)	51 (1.10)	37 (1.60)	12 (1.55)	13.801	0.017	–
HPV35	104	0.68 (0.55–0.81)	2 (0.37)	15 (0.53)	32 (0.77)	28 (0.60)	17 (0.73)	10 (1.29)	6.994	0.221	–
HPV82	102	0.67 (0.54–0.80)	9 (1.67)	15 (0.53)	21 (0.51)	33 (0.71)	21 (0.91)	3 (0.39)	13.555	0.019	–
HPV45	89	0.58 (0.46–0.71)	2 (0.37)	12 (0.42)	21 (0.51)	28 (0.60)	22 (0.95)	4 (0.52)	7.496	0.186	–
HPV73	49	0.32 (0.23–0.41)	2 (0.37)	6 (0.21)	8 (0.19)	19 (0.41)	11 (0.47)	3 (0.39)	6.146	0.292	–
LR-HPV											
HPV42	503	3.30 (3.02–3.59)	25 (4.64)	63 (2.23)	139 (3.36)	148 (3.20)	101 (4.36)	27 (3.49)	21.665	0.001	2.41% (0.55–4.27)
HPV81	329	2.16 (1.93–2.39)	8 (1.48)	53 (1.87)	72 (1.74)	110 (2.38)	63 (2.72)	23 (2.97)	12.533	0.028	–
HPV43	268	1.76 (1.55–1.97)	13 (2.41)	53 (1.87)	58 (1.40)	87 (1.88)	39 (1.68)	18 (2.33)	6.460	0.264	–
HPV44	218	1.43 (1.24–1.62)	16 (2.97)	33 (1.17)	49 (1.19)	69 (1.49)	39 (1.68)	12 (1.55)	13.434	0.020	–
HPV11	207	1.36 (1.18–1.54)	24 (4.45)	33 (1.17)	49 (1.19)	59 (1.27)	35 (1.51)	7 (0.90)	42.014	<0.001	3.29% (1.50–5.07)
HPV6	155	1.02 (0.86–1.18)	28 (5.19)	26 (0.92)	27 (0.65)	32 (0.69)	33 (1.42)	9 (1.16)	107.910	<0.001	4.28% (2.37–6.18)
HPV83	75	0.49 (0.38–0.60)	2 (0.37)	9 (0.32)	21 (0.51)	22 (0.48)	18 (0.78)	3 (0.39)	5.965	0.310	–

For the 24 HPV genotypes compared across six age groups, the corrected significance threshold was set at *α* = 0.05/24 = 0.0021. Genotype-specific prevalence was calculated using the total number of women tested (*N* = 15,224) as the denominator. Rate difference = HPV prevalence in the ≤24 years group − HPV prevalence in the 25–34 years group. For the remaining genotypes, comparisons did not reach the Bonferroni-corrected significance threshold (*p* < 0.0021); therefore, rate differences were not reported.

## Discussion

This study revealed that the overall HPV prevalence among gynecological outpatients at our hospital in Xuzhou from 2023 to 2025 was 32.08%. It is important to emphasize that these data reflect HPV positivity at a single time point, and because follow-up data were not available, we could not distinguish transient infections from persistent infections. Therefore, these prevalence estimates should not be directly interpreted as reflecting the burden of clinically significant, persistent HPV infection or cervical cancer risk. The observed prevalence was higher than the global female HPV prevalence (11.7%) (10) and the overall prevalence among Chinese women (17.7%) (7), and also exceeded the rates reported in several areas within Jiangsu Province, such as Zhangjiagang (19.58%) (11) and Suzhou (10.2% for HR-HPV) (12). This discrepancy may be attributable to multiple factors. First, all participants in this study were gynecological outpatients, whereas most of the comparative studies above mainly enrolled women undergoing health check-ups or community-based screening; it is plausible that outpatients with gynecological symptoms or cervical lesion-related complaints may have a higher HPV detection rate. Second, the study period (2023–2025) fell in the later phase of the rising national HPV prevalence trend. A systematic review encompassing over 1.07 million Chinese women showed that the national HPV prevalence during 2017–2021 (26.06%) was significantly higher than that during 2010–2016 (23.20%) (13), and this temporal increase may partially explain the relatively high prevalence observed in our study. In addition, our assay detected 24 HPV genotypes, and different studies used different detection kits; theoretically, kits covering more genotypes tend to yield higher positive rates. These explanations for the relatively high prevalence are hypotheses that cannot be directly verified with the data available in this study and require further investigation. A recent systematic review in healthy Chinese women reported a single-infection prevalence of approximately 10.4% and a multiple-infection prevalence of approximately 4.7% (14). In our study, the single-infection rate was 20.93% and the multiple-infection rate was 11.15% (6.88% for double, 2.54% for triple, 1.05% for quadruple, and 0.67% for ≥quintuple genotypes), both rates and the proportion of multiple infections being higher than the above reports. This is likely related to the heavier disease burden in gynecological outpatients compared with healthy populations; symptomatic women or those with lesions are more susceptible to concurrent infection with multiple HPV types. Moreover, single HR-HPV infection (16.48%) predominated in our study, while multiple high-risk infections (5.40%) and mixed high and low-risk infections (5.42%) were comparable, suggesting that HR-HPV genotypes constitute the main component of infections among gynecological outpatients at our hospital, which is consistent with most domestic studies.

Regarding genotype distribution, the five most prevalent genotypes in our study were HPV52 (5.58%), HPV16 (4.93%), HPV58 (4.49%), HPV53 (3.54%), and HPV42 (3.30%), with the top five high-risk genotypes being HPV52, HPV16, HPV58, HPV53, and HPV56. Without cytology/pathology, our frequent HR-HPV findings (52, 16, 58, 53, 56) do not confirm cervical lesion risk; prevalence does not imply oncogenicity and further genotype-specific studies are required. This genotypic spectrum is highly consistent with the national trend during 2017–2021, the five most common HPV genotypes among Chinese women were HPV52, HPV16, HPV58, HPV53, and HPV51 (13); a pooled analysis covering over 2.72 million Chinese women also confirmed that HPV52 is the most common oncogenic type, followed by HPV58 and HPV16 (7). Data from within Jiangsu Province similarly indicated that HPV52 was the most prevalent high-risk genotype, with an infection rate of 4.14% in Zhangjiagang (11). Notably, HPV16 is generally the dominant genotype worldwide, but in China and East Asian populations, the prevalence of HPV52 often exceeds or approaches that of HPV16; this regional difference may be related to variations in genetic background, sexual behavior patterns, and viral evolution across populations (15). Among low-risk genotypes, HPV42 (3.30%) was the most prevalent in our study, higher than HPV81 (2.16%) and HPV43 (1.76%). Domestic studies have paid relatively limited attention to low-risk HPV42, but a report from Changsha, Hunan, showed an HPV42 prevalence of 1.75% (16), and in Weifang, Shandong, HPV42 was the most common low-risk type (2.19%) (17); meanwhile, HPV81 was reported as the predominant low-risk type in Yueyang, Hunan (2.1%) (18) and Jingzhou, Hubei (3.33%) (19), suggesting that low-risk HPV genotype distribution also exhibits regional variation. It is important to emphasize that the relatively high prevalence of LR-HPV genotypes (HPV42, 81, and 43) in our study population reflects their frequency of detection rather than their oncogenic potential. Unlike HR-HPV genotypes, LR-HPV types are not established as causal agents of cervical cancer, and their clinical significance lies primarily in their association with benign genital warts and other non-malignant conditions. Therefore, the prevalence data presented here should not be interpreted as indicating that these genotypes contribute substantially to the cervical cancer burden. Among the top five genotypes in our study, HPV52, 58, and 16 are covered by current vaccines, but high-risk types such as HPV53 and 56, as well as low-risk types including HPV42, 81, and 43, are not covered by existing vaccines, indicating a protective gap. However, a high prevalence of HR-HPV does not equate to oncogenic risk, and vaccine target selection requires additional evidence including persistence, association with precancer/cancer, and population-level benefit. Currently available vaccines cover only 7 high-risk HPV types and 2 low-risk HPV types, resulting in a limited protective spectrum (20). Our study found that non-vaccine-covered genotypes (HPV53, 56, 42, 81, and 43) exhibited high infection prevalence. Although epidemiological observations alone do not constitute sufficient evidence for inclusion in future vaccines, these findings may support further surveillance and etiological research on these genotypes.

Analysis of HPV prevalence across different years showed that from 2023 to 2025, the overall HPV prevalence among gynecological outpatients in Xuzhou exhibited a continuous upward trend, increasing from 27.57 to 36.63%, and all infection patterns and infection types, except for multiple low-risk infections, increased significantly over the years. The annual increasing trend in infection rates may be affected by variations across different years, such as age distribution, healthcare-seeking behavior, referral patterns, screening practices and other factors. After adjusting for age in this study, the upward trend in infection rates persisted. Further research is needed to determine whether the observed annual trend is driven by differences in other characteristics of the study populations over time. This trend aligns with the direction reported in multiple national and regional studies, although the magnitude of increase was more pronounced in our study. A nationwide observational study covering 33,750,381 Chinese women from 2015 to 2025 reported that HPV prevalence rose from 7.75 to 22.48% (21), which is consistent with the upward direction observed here. In Shanghai, the high-risk HPV prevalence dropped to a low of 12.25% during the COVID-19 epidemic period (2020–2022), rebounded to 17.54% in 2023, and further increased to 19.47% in early 2024 (22), suggesting that COVID-19 control measures had a significant impact on HPV epidemiology. The steeper increase in our study compared with national and other regional data may result from the combined effects of several factors. First, all participants in our study were gynecological outpatients, whereas both the national study and the Chengdu study included a large proportion of health check-up populations; outpatients with symptoms or cervical lesion complaints have a higher HPV detection rate. Second, disruptions in HPV screening and vaccination services during the COVID-19 pandemic may have led to the accumulation of previous infections, and the post-pandemic surge in healthcare-seeking behavior further elevated detection rates. Third, our observation period (2023–2025) coincided with an accelerating phase of the national upward HPV prevalence trend. National data showed that the HPV prevalence during 2017–2021 (26.06%) was already significantly higher than that during 2010–2016 (23.20%), with particularly notable increases in genotypes HPV52, 58, 53, 56, 39, 51, 59, 66, and 68 (13). These findings may have potential implications for regional cervical cancer prevention strategies, such as strengthening HPV vaccination and screening programs. However, our study was not designed to evaluate the effectiveness of these interventions, and these recommendations should be considered preliminary and hypothesis-generating rather than definitive policy guidance.

Dynamic analysis of genotype distribution across different years revealed that HPV52, 16, and 58 consistently ranked among the top three over the three-year period, and this stable dominance pattern is highly consistent with recent epidemiological characteristics in China. A national study of 1,191,312 women from 2020 to 2024 showed that HPV52 (3.36%), HPV16 (2.56%), and HPV58 (2.46%) were the most prevalent genotypes (23). Of note, HPV52 was the top genotype in our study, whereas national data indicated a significant decline in HPV16 during 2017–2021 compared with 2010–2016 (*p < 0.05*) (13); together, these findings suggest that a potential change in the relative prevalence of HPV16 and HPV52, although longer-term surveillance is needed to determine whether this represents true genotype replacement. Among low-risk genotypes in our study, HPV42 and 81 consistently ranked in the top two, with HPV42 being the most prevalent low-risk type, warranting attention to its clinical significance.

Analysis of HPV prevalence by age group among gynecological outpatients in Xuzhou showed that the overall prevalence exhibited a “bimodal” distribution, first declining and then rising, with the highest rate in the ≤24-year group (42.30%), followed by the 55–64 years group (37.29%) and the ≥65 years group (36.43%), and the lowest in the 25–34-year group (29.05%). This age distribution pattern is highly consistent with multiple domestic studies. A study of 181,705 women in Chengdu showed a bimodal distribution, with the first peak in the <20-year group (42.97%) and the second in the 60–69-year group (37.56%) (24); a study of 123,808 women in Bengbu also presented a bimodal distribution, with rates of 17.39% in the <25-year group and 21.14% in the 60–64-year group (25). A study of 14,492 outpatients in Wuhan showed peaks at <25 years (22.88%) and >56 years (24.26%) (26). A Beijing study indicated that HR-HPV was more prevalent in women aged <25 years and 55–59 years (27). Similar to the overall prevalence, infection patterns and infection types across age groups also exhibited a bimodal distribution, with the first peak in the ≤24-year group and the second peak in the older age groups. Because the HPV prevalence was similarly high in the 55–64-year and ≥65-year groups, the specific age group corresponding to the second peak varied slightly across different infection patterns and types. Although the specific age nodes and absolute prevalence rates for different infection patterns and types vary across countries and regions, the overall epidemiological pattern of “youth peak—middle-age trough - middle-to-older age rebound” appears relatively stable. The first peak may be partially explained by active sexual activity in young women, immature cervical epithelial barriers, and higher risk of new HPV exposure. The second peak may be explained by age-related immune senescence and declining estrogen levels in middle-aged and older women, leading to compromised genital mucosal barriers, reactivation of previously latent HPV, and consequent viral persistence and reinfection. While the observed bimodal age distribution is consistent with previous reports, the underlying mechanisms cannot be directly verified with the data available in this study. These factors should therefore be considered as possible explanations, but they require confirmation in future studies incorporating behavioral, hormonal, vaccination, and longitudinal infection data. These findings suggest the potential value of a dual-track strategy of “vaccination plus standardized screening”: promote HPV vaccination among adolescents to reduce the first peak, and strengthen systematic screening and follow-up for middle-aged and older women to control the second peak, which should be confirmed in future studies with clinical outcome data, such as cytological, histopathological, and persistence data. These findings are descriptive and cannot establish causality; therefore, the proposed dual-track strategy is a possible implication that requires confirmation.

Analysis of genotype distribution across age groups revealed that in the ≤24-year group, the predominant low-risk genotypes were HPV6, 42, and 11, whereas in other age groups they were HPV42, 81, and 43; HPV6 and 11 showed the highest positive rates in the ≤24-year group (5.19 and 4.45%, respectively), which is consistent with the higher incidence of condyloma acuminata in young sexually active populations (28).

Several limitations of this study should be acknowledged. First, this was a single-center retrospective study conducted among women attending the gynecological outpatient clinic of the Affiliated Hospital of Xuzhou Medical University. This single-center outpatient setting inherently limits the generalizability of our findings, because the study population consisted of women seeking care due to symptoms or screening needs—who are likely to have a higher HPV prevalence than the general female population. Therefore, our results should not be extrapolated to the general female population of Xuzhou. Second, only baseline HPV testing data were available, and no follow-up information was collected for HPV-positive women. This is a critical limitation, as it precludes the distinction between transient infections—which are common and typically cleared by the host immune system—and persistent infections, which are the true precursors of cervical cancer and precancerous lesions. Consequently, our prevalence estimates cannot be directly translated into the risk of clinically significant cervical disease, and any inference regarding cervical cancer risk should be made with caution. Third, cervical cytology or pathology results were not included, precluding assessment of the association between different HPV genotypes and the severity of cervical lesions. Fourth, behavioral information (such as sexual history, contraceptive methods, smoking status), vaccination history, and socioeconomic factors were not collected, limiting in-depth analysis of influencing factors for HPV infection. Fifth, the age-adjusted analyses were not performed for all subgroups (e.g., LR + LR infection) or all genotypes because of limited case numbers; these results should therefore be interpreted with caution. Future studies should adopt multicenter, prospective cohort designs, incorporate cytological and pathological data, and include behavioral and vaccine-related information to more comprehensively evaluate the epidemiological characteristics and clinical implications of HPV infection in Xuzhou.

In conclusion, the overall HPV prevalence among women attending the gynecological outpatient clinic of the Affiliated Hospital of Xuzhou Medical University was 32.08%, which is at a relatively high level, with single high-risk infection being the predominant pattern, and the prevalence showed a continuous upward trend from 2023 to 2025. HPV52, 16, 58, 53, and 56 were the major high-risk genotypes, while HPV42, 81, and 43 were the major low-risk genotypes. The age distribution exhibited a bimodal distribution, with women aged ≤24 years and 55–64 years being the high-risk groups. Our findings provide important baseline epidemiological data on HPV prevalence and genotype distribution among gynecological outpatients in Xuzhou. These data may have preliminary implications for regional cervical cancer prevention strategies. In particular, the high prevalence of non-vaccine-covered genotypes (HPV53, 56, 42, 81, and 43) warrants continued surveillance and further investigation of their clinical significance. Additionally, the observed age-specific distribution supports the potential value of targeted screening strategies for younger and older women. However, it should be emphasized that our findings reflect HPV prevalence at a single time point rather than persistent infection; because follow-up data were not available, these estimates cannot be directly translated into cervical cancer risk. These implications should be considered preliminary and require confirmation in future studies incorporating cytological, pathological, and longitudinal follow-up data.

## Data Availability

The original contributions presented in the study are included in the article/Supplementary material, further inquiries can be directed to the corresponding authors.
