# Supplementary material for: Prevalence and genotype distribution of human papillomavirus among 15,224 gynecological outpatients in Xuzhou, China, during 2023–2025
Source: Front Public Health. 2026 Oct 5;14:1969653. doi: 10.3389/fpubh.2026.1969653 (PMC13677893; doi:10.3389/fpubh.2026.1969653)
Supplement: Supplementary file 1 [file Supplementary_file_1.pdf]

SUPPLEMENTARY FIGURE S1 Flow diagram of the study participants

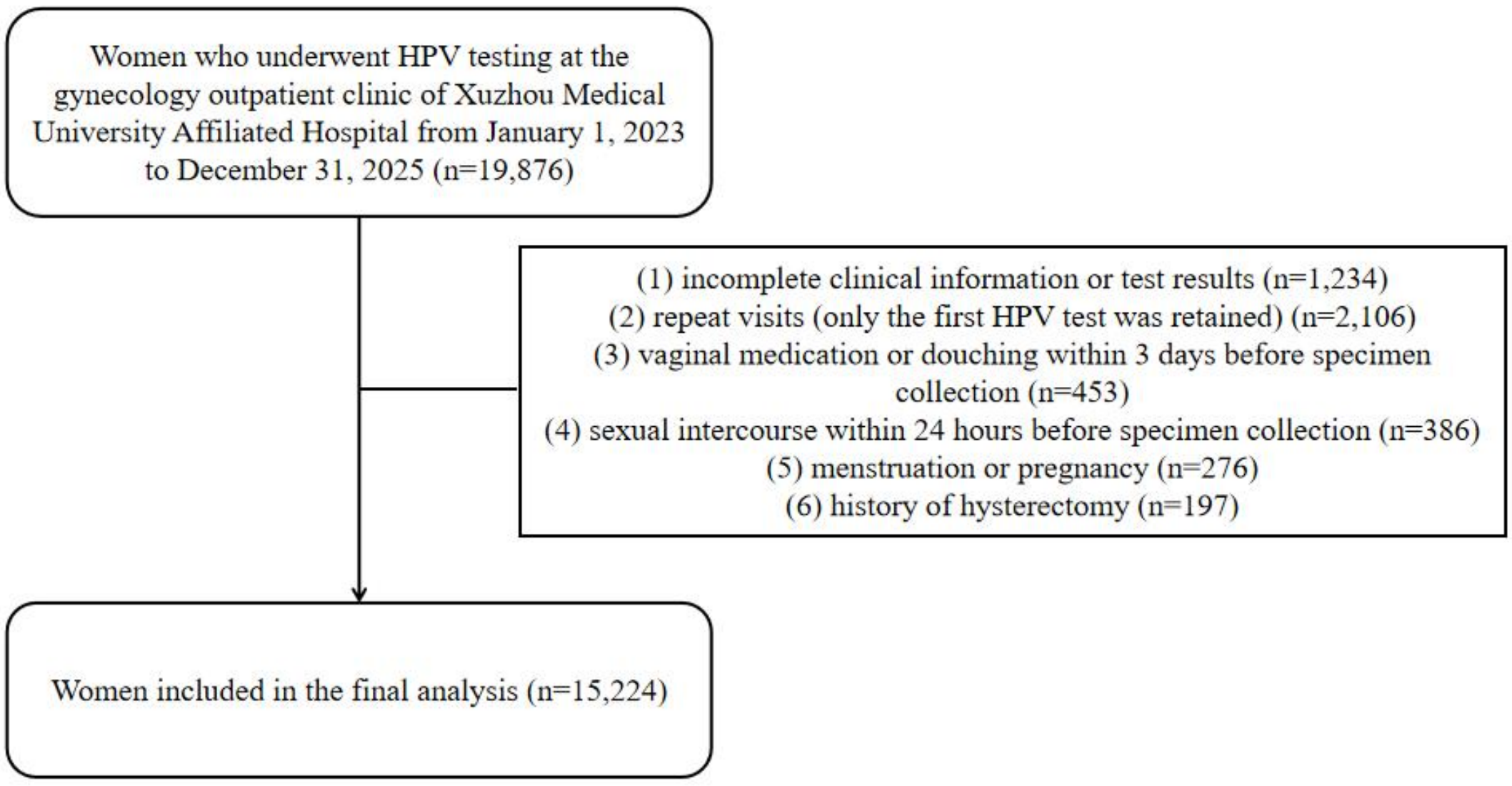

SUPPLEMENTARY TABLE S1 Distribution of study participants by age group and year.

Age group (years)	2023（*N*=6, 286） *n*（%）	2024（*N*=4, 671） *n*（%）	2025（*N*=4, 267） *n*（%）
≤24	235（3. 74）	146（3. 13）	158（3. 70）
25 – 34	1, 254（19. 95）	867（18. 56）	709（16. 62）
35 – 44	1, 677（26. 68）	1, 268（27. 15）	1, 188（27. 84）
45 – 54	2, 013（32. 02）	1, 380（29. 54）	1, 238（29. 01）
55 – 64	821（13. 06）	743（15. 91）	753（17. 65）
≥65	286（4. 55）	267（5. 72）	221（5. 18）

N, total number of participants in that year; *n*, number of participants in each age subgroup for that year; Percentage (%) = $n/N \times 100$. Percentages may not sum to 100% due to rounding.

SUPPLEMENTARY TABLE S2 Age-adjusted multivariable logistic regression analysis of HPV prevalence by study year (2024 vs 2023 and 2025 vs 2023) for overall HPV infection, infection patterns, infection types, and the eight genotypes that remained significant after Bonferroni correction.

HPV infection / genotype	Adjusted OR (2024 vs 2023) (95% CI)	*P*	Adjusted OR (2025 vs 2023) (95% CI)	*P*
Positive cases	1.346 (1.240–1.462)	<0.001	1.501 (1.380–1.632)	<0.001
**Infection patterns**				
Single	1.257 (1.144–1.381)	<0.001	1.344 (1.221–1.479)	<0.001
Double	1.329 (1.142–1.547)	<0.001	1.353 (1.159–1.580)	<0.001
Triple	1.228 (0.959–1.574)	0.104	1.381 (1.079–1.765)	0.010
Quadruple	1.203 (0.795–1.820)	0.382	2.101 (1.450–3.047)	<0.001
≥Quintuple	1.428 (0.872–2.338)	0.156	1.764 (1.094–2.843)	0.020
**Infection types**				
Single HR	1.176 (1.061–1.304)	0.002	1.252 (1.128–1.391)	<0.001
Single LR	1.454 (1.204–1.756)	<0.001	1.533 (1.267–1.854)	<0.001
HR + HR	1.195 (1.007–1.419)	0.042	1.352 (1.140–1.603)	0.001
HR + LR	1.519 (1.277–1.808)	<0.001	1.666 (1.399–1.983)	<0.001
LR + LR	—	—	—	—
**Genotypes**				
HPV52	1.178 (0.993–1.396)	0.060	1.402 (1.186–1.657)	<0.001

HPV56	1.530 (1.191–1.965)	0.001	1.589 (1.233–2.047)	<0.001
HPV39	1.424 (1.104–1.836)	0.006	1.649 (1.283–2.121)	<0.001
HPV59	1.498 (1.125–1.994)	0.006	1.747 (1.317–2.316)	<0.001
HPV18	1.035 (0.699–1.531)	0.863	1.861 (1.315–2.632)	<0.001
HPV68	1.512 (1.170–1.955)	0.002	1.460 (1.121–1.901)	0.005
HPV43	1.822 (1.355–2.451)	<0.001	1.652 (1.212–2.252)	0.001
HPV11	2.657 (1.791–3.943)	<0.001	3.789 (2.595–5.534)	<0.001

Age-adjusted multivariable logistic regression was performed with study year (reference: 2023) and age group (reference: 25–34 years) as categorical independent variables. Adjusted odds ratios (ORs) with 95% confidence intervals (CIs) are shown. LR+LR infection was not analyzed due to limited positive cases ($n = 50$).
